# Pathogenic Variants in *RNPC3* are Associated with Hypopituitarism and Primary Ovarian Insufficiency

**DOI:** 10.1016/j.gim.2021.09.019

**Published:** 2021-11-30

**Authors:** Leyla Akin, Karine Rizzoti, Louise C Gregory, Beatriz Corredor, Polona Le Quesne Stabej, Hywel Williams, Federica Buonocore, Stephane Mouilleron, Valeria Capra, Sinead M McGlacken-Byrne, Gabriel Á Martos-Moreno, Dimitar N. Azmanov, Mustafa Kendirci, Selim Kurtoglu, Jenifer P. Suntharalingham, Christophe Galichet, Stefano Gustincich, Velibor Tasic, John C. Achermann, Andrea Accogli, Aleksandra Filipovska, Anatoly Tuilpakov, Mohamad Maghnie, Zoran Gucev, Z. Burcin Gonen, Luis A Pérez-Jurado, Iain Robinson, Robin Lovell Badge, Jesús Argente, Mehul T Dattani

**Affiliations:** 1 Department of Paediatric Endocrinology, Faculty of Medicine, Ondokuz Mayıs University, Samsun, Turkey; 2 Department of Paediatric Endocrinology, Faculty of Medicine, Erciyes University, Kayseri, Turkey; 3 The Francis Crick Institute, London, UK; 4 Genetics and Genomic Medicine Research and Teaching Department, UCL Great Ormond Street Institute of Child Health, London, UK; 5 Hospital Infantil Universitario Niño Jesús, Departments of Paediatrics and Paediatric Endocrinology, Madrid, Spain; 6 GOSgene, Genetics and Genomic Medicine, Research and Teaching Department, UCL Great Ormond Street Institute of Child Health, London, UK; 7 Department of Molecular Medicine and Pathology, University of Auckland, Auckland, New Zealand; 8 Genetics and Genomic Medicine, School of Medicine, Cardiff University, UK; 9 Structural Biology Science Technology Platforms, The Francis Crick Institute, 1 Midland Road, London, UK; 10 Unit of Medical Genetics, IRCCS Istituto Giannina Gaslini, Genova, Italy; 11 Universidad Autónoma de Madrid, Department of Paediatrics, Madrid, Spain; 12 CIBER Fisiopatología Obesidad y Nutrición (CIBERobn), Instituto de Salud Carlos III, Madrid, Spain; 13 Centre of Medical Research, The University of Western Australia and Harry Perkins Institute for Medical Research, Perth, Western Australia, Australia; 14 Department of Diagnostic Genomics, PathWest, QEII Medical Centre, Perth, Western Australia, Australia; 15 Istituto Italiano di Tecnologia – IIT Via Enrico Melen 83, Building B, Genova, Italy; 16 University Children’s Hospital, Medical School, Skopje, North Macedonia; 17 Department of Neurosciences, Rehabilitation, Ophthalmology, Genetics, Maternal and Child Health (DINOGMI) – University of Genova, Genova, Italy; 18 Telethon Kids Institute, Northern Entrance, Perth Children's Hospital, 15 Hospital Avenue, Nedlands, Western Australia, Australia; 19 Department of Endocrine Genetics, Research Centre for Medical Genetics, Ulitsa Moskvorechye, Moscow, Russian Federation; 20 Department of Inherited Endocrine Disorders, Endocrinology Research Centre, Dmitriya Ulianova Street, Moscow, Russian Federation; 21 Department of Paediatrics, IRCCS Istituto Giannina Gaslini, Genova, Italy; 22 Oral and Maxillofacial Surgery, Genome and Stem Cell Center, Erciyes University, Kayseri, Turkey; 23 Genetics Unit, Universitat Pompeu Fabra, Hospital del Mar Research Institute (IMIM) and Centro de Investigación Biomédica en Red de Enfermedades Raras (CIBERER), Barcelona, Spain; 24 South Australian Health and Medical Research Institute (SAHMRI), The University of Adelaide, Adelaide, SA, Australia; 25 IMDEA Food Institute, CEI UAM + CSIC, Carretera de Cantoblanco 8, Madrid, Spain; 26 Department of Paediatric Endocrinology, Great Ormond Street Hospital for Children, London, UK

**Keywords:** Primary ovarian insufficiency, hypopituitarism, growth hormone deficiency, minor spliceosome, U12-type spliceosome

## Abstract

**Purpose:**

We aimed to investigate the molecular basis underlying a novel phenotype including hypopituitarism associated with POI.

**Methods:**

We used NGS to identify variants in all pedigrees. Expression of *Rnpc3/RNPC3* was analysed by in situ hybridization on murine/human embryonic sections. Crispr/Cas9 was used to generate mice carrying the p.L483F pathogenic variant in the conserved murine Rnpc3 RRM2 domain.

**Results:**

We described 15 patients from nine pedigrees with biallelic mutations in *RNPC3,* encoding a specific protein component of the minor spliceosome, associated with a hypopituitary phenotype including severe GHD, hypoprolactinaemia, variable TSH deficiency and anterior pituitary hypoplasia. POI was diagnosed in eight of nine affected females whilst males had normal gonadal function. Additionally, two affected males displayed normal growth off GH treatment, despite severe biochemical GHD. In both mouse and human embryos, *Rnpc3/RNPC3* was expressed in the developing forebrain including the hypothalamus and Rathke’s pouch. Female *rnpc3* mutant mice displayed a reduction in pituitary GH content, but with no reproductive impairment in young mice. Male mice exhibited no obvious phenotype.

**Conclusion:**

Our findings suggest novel insights into the role of RNPC3 in female-specific gonadal function and emphasize a critical role of the minor spliceosome in pituitary and ovarian development and function.

## Introduction

Primary ovarian insufficiency (POI), characterized by amenorrhoea with elevated gonadotropin concentrations, includes a spectrum ranging from 46,XX gonadal dysgenesis to premature menopause. Non-syndromic POI occurs in 1% of women; however, early-onset forms manifesting as primary amenorrhoea with absent pubertal development affect approximately 1:100000 females.^1^ To date, more than 50 genes have been associated with POI^2^. Overall, known genetic causes, including X chromosomal abnormalities, account for 20-25% of cases.^3–11^

Pre-mRNA splicing is an essential step in gene expression in all eukaryotes. Two distinct splicing machineries, the major (U2-dependent) and minor (U12-dependent) spliceosomes, recognize and excise either the major (U2-type) or minor (U12-type) class of introns, respectively. Minor introns constitute ~0·35% of all human introns and are present in approximately 700–800 genes.^12^ *RNPC3* encodes the U11/U12-65K protein, a component of the U12-dependent spliceosome.^13^

Here, we describe 15 patients from nine pedigrees harbouring variants in *RNPC3* with a hypopituitary phenotype including severe growth hormone deficiency (GHD, previously described in ref 14), hypoprolactinaemia, variable thyrotropin (TSH) deficiency and anterior pituitary hypoplasia (APH). POI was diagnosed in eight of nine affected females, whilst all males had normal gonadal function. We performed genetic and functional studies to elucidate the molecular basis underlying this novel phenotype.

## Materials And Methods

### Participants

**Figure 1:**
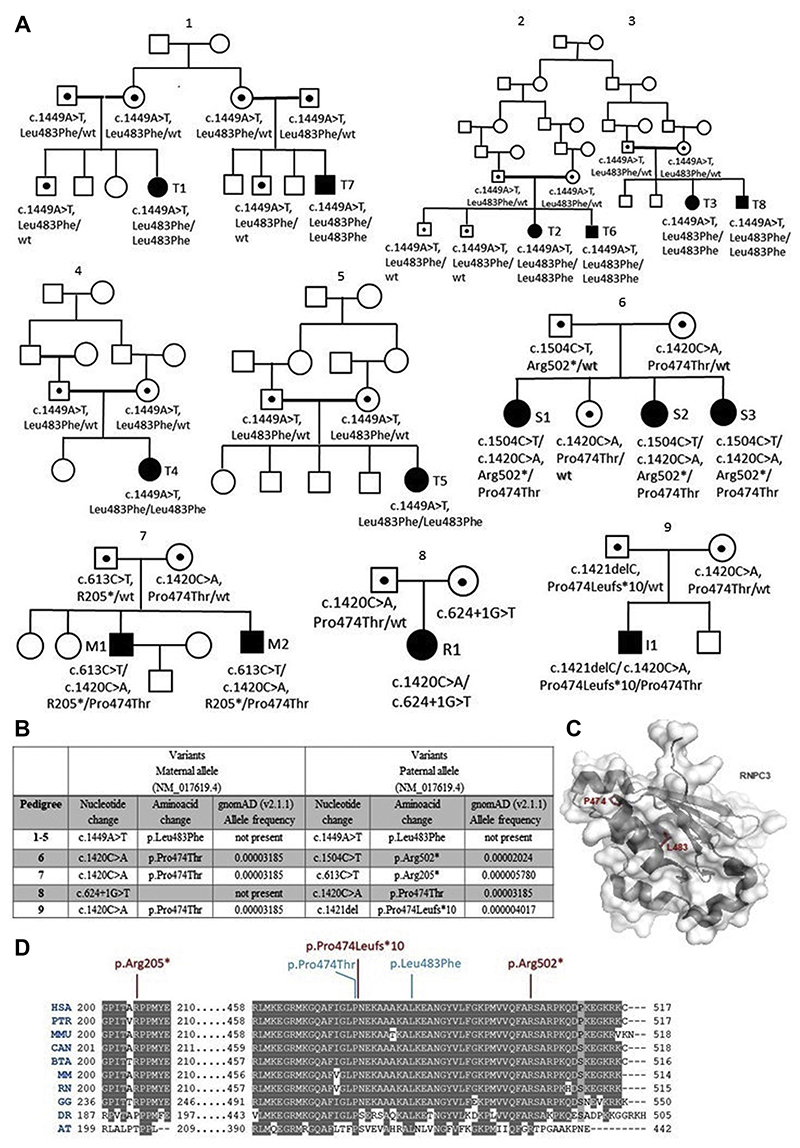
A) Pedigrees 1-9 harbouring the *RNPC3* variants. All affected patients are homozygous in pedigrees 1-5 and compound heterozygotes in pedigrees 6-9 for the *RNPC3* variants shown under each symbol while the unaffected parents are heterozygotes for the relevant variants. Shaded squares and circles indicate affected family members, squares for males, circles for females. Heterozygote members are indicated with a dot in each symbol. Shapes joined by thick lines indicate consanguinity between those individuals. Pedigree numbers are given at the top of each pedigree, and each affected subject is marked with the relevant patient IDs relating to the text and tables. B) The pathogenic variants found in *RNPC3* in pedigrees 1-9. C) The structure of the C-Terminal RNA recognition motif of the U11/U12 65K protein (PDB-ID: 3EGN) displayed in dark grey cartoon and transparent surface. Residues P474 and L483, discussed in the text, are displayed in red sticks. D) The conservation of substitutions, RNPC3 (p.L483F), (p.P474T), (p.R502X), (p.R205X) and (p.P474LfsX10) across multiple species. (HSA: *Homo sapiens*; PTR: *Pan troglodytes*; MMU: *Macaca mulatta*; CAN: *Canis lupus*; BTA: *Bos taurus*; MM: *Mus musculus*; RN: *Rattus norvegicus*; GG: *Gallus gallus*; DR: *Danio rerio*; AT: *Arabidopsis thaliana*)

Five first-degree consanguineous pedigrees, living in geographically close areas in Turkey, included eight (5F, 3M) Turkish patients (T1-T8). Pedigree 6 included three previously described^14,15^ Romanian-origin Spanish sisters (S1-S3). Pedigree 7 included two Turkish-origin Macedonian brothers (M1 and M2); Pedigree 8 Russian patient R1 and pedigree 9 Albanian-origin Italian patient I1. Pedigrees 6-9 were not consanguineous (Fig.1A).

### Genetic studies

Next Generation Sequencing (NGS) was performed in all pedigrees, followed by Sanger sequencing to confirm NGS-discovered variants, and segregation of identified variants in unaffected family members.

### *In situ* hybridization studies

*In situ* hybridization (ISH) studies were performed in human and mouse embryos and adult mice to analyse *RNPC3* expression in the hypothalamo-pituitary (HP) region and ovaries.

### Mouse models

**Figure 2:**
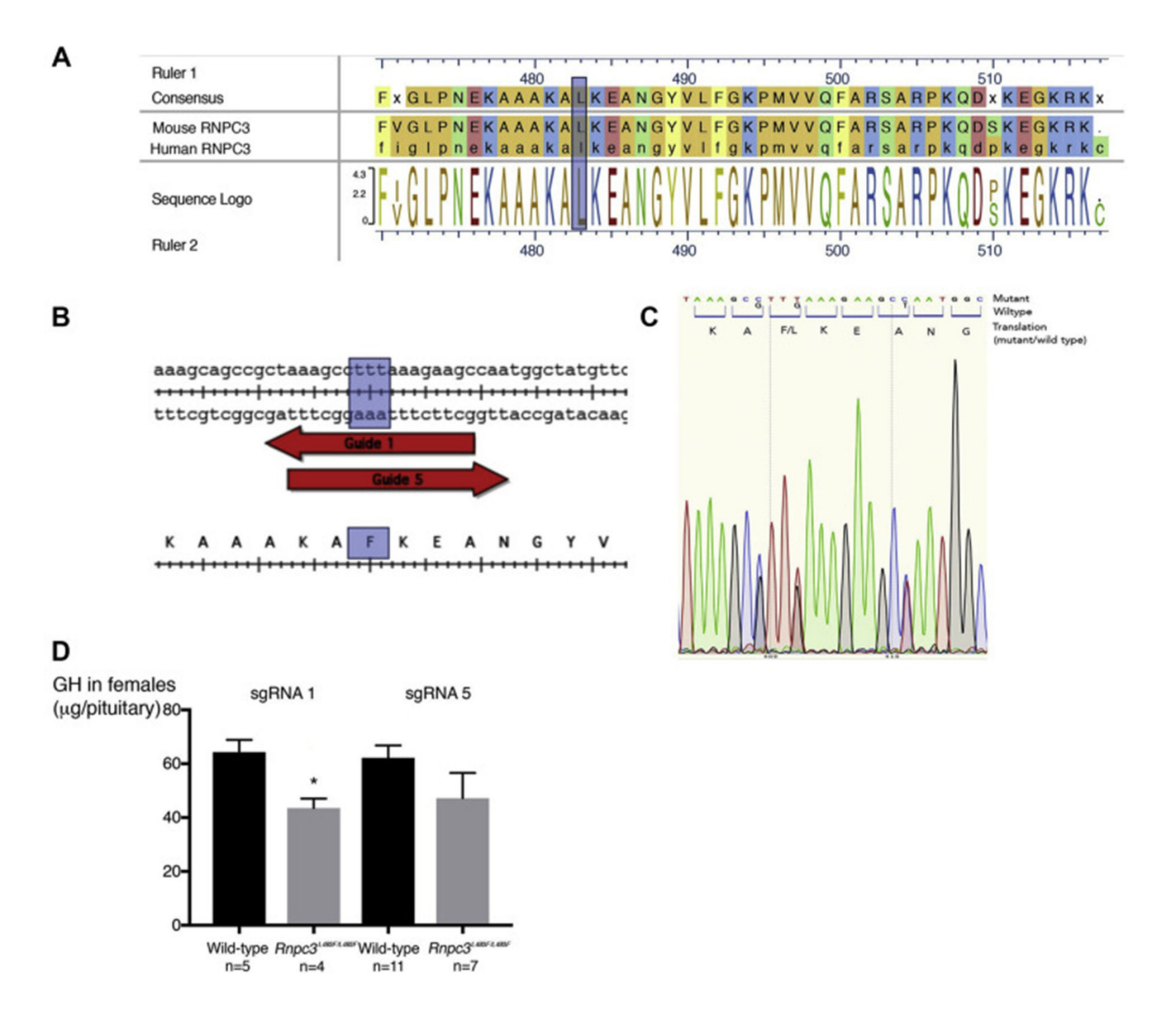
Murine data including the methods and the results. A) Alignment of the mouse and human RNPC3 protein sequences. The leucine residue at position 483 that is mutated in patients and the surrounding region are conserved between mouse and human. B) Two single guide RNA were designed where the leucine residue (ttg) was mutated into phenylalanine (ttt). C) Chromatogram of a mouse heterozygous Rnpc3 mutant. Sanger sequencing showing mutated nucleotides giving rise to the desired mutation (L to F) and the introduction of two silent mutations to avoid subsequent cutting by the sgRNA. D) RIA performed on female pituitaries comparing homozygous mutants and wild-type littermate controls. A decrease in GH levels was observed in both sgRNA1 and 5 strains, but it was only statistically significant in sgRNA1 (64.28±4.6 SEM in controls versus 43.4±3.5 SEM in mutants, p=0.016).

Using Crispr/Cas9 technology, we generated mice carrying the p.L483F variant in the conserved RRM2 domain of murine Rnpc3. We also generated mice harbouring the p.P350R variant in *Prmt6* (Fig.2, Fig.S1).

The methodology of the genetic, ISH and mouse studies is detailed in the Appendix.

## Results

### Clinical assessment

#### Growth patterns

All patients were born with a normal birthweight but showed severe postnatal growth retardation (heights -4·1 to -8·9 SDS at presentation) with typical features of GHD (Table-1). Although IGF-1 and peak GH to provocation tests were undetectable at age 9.7 years (y), patient T6 showed growth without GH until age 15y, when the growth velocity decreased to 4 cm/year and rhGH therapy was started. He did not achieve his target adult height (Fig.S2A).

Patients M1 and M2 (Pedigree 7) presented with severe GHD at ages 28 and 20 months, respectively. Rapid growth ensued in the first two years on treatment, with heights on the 30th and 40th percentiles on the growth curve. Subsequently, growth continued without GH treatment during puberty. Following cessation of rhGH therapy (age 9y), the eldest boy (M1) had a 36 cm pubertal growth spurt, achieving a final height exceeding target height range. The younger brother (M2) achieved a pubertal growth spurt of around 30 cm following GH therapy discontinuation at 5.5y, but his final height was at the lower end of the target height range (Fig.S2A). Retesting GH pituitary reserve showed low peak GH concentrations (<0·3ng/ml).

Growth responses to rhGH therapy were excellent in all patients (Table-1).

#### Anterior pituitary function

In all patients, basal IGF-I and IGFBP-3 concentrations were almost undetectable as were peak GH concentrations to provocation. Basal serum cortisol and ACTH concentrations were normal. In pedigrees 1-5, there was mild thyroid dysfunction (low serum free thyroxine (fT4) with a normal TSH, or normal fT4 with a slightly elevated TSH) in all patients, and L-T4 replacement was required in two of them. In patient I1, central hypothyroidism was detected at age 14·5y requiring L-T4 replacement. Prolactin (PRL) concentrations were low in most patients (Table-1).

#### Brain MRI

Brain MRI revealed APH in 13 of 15 patients (Table-1). The posterior pituitary was eutopic in all patients. Patient I1 had an irregular sellar floor, mild hypoplasia of the splenium of corpus callosum and mild cerebellar tonsil herniation on MRI.

#### Ovarian/Testicular phenotype

Pedigree 1-5: The proband in Pedigree 1 (the eldest girl in the cohort) had elevated serum FSH with a normal LH concentration (24·8 and 0·7IU/L, respectively) on presentation at age 8y. Spontaneous puberty ensued at age 14y, with FSH and LH concentrations of 86·6IU/L and 32·1U/L, respectively. Breast development progressed slowly and arrested at Tanner stage 3 with undetectable serum oestradiol concentrations. The other four female Turkish patients also had increased gonadotropin concentrations according to age; relatively decreased during the ages of 3-6 years and progressively elevated thereafter. Pelvic ultrasonography showed a small uterus with undetectable or small ovaries and no follicles in all five girls (age range at investigation: 3-21 years, Table-2). Pubarche started at normal age, but the hair was sparse in all female patients. Menarche did not occur in any of the girls. All female patients had 46,XX karyotype, with no stigmata of Turner syndrome. Oestrogen replacement therapy resulted in normal breast development; menarche with regular menstruation was achieved by cyclic oestrogen/progestagen therapy.

In pedigree 6, patient S1 was prepubertal at age 15·4y at the time of GHD diagnosis, with baseline FSH and LH concentrations of 30·3IU/L and 6·7IU/L respectively, peaking after Gonadotropin-releasing hormone (GnRH) stimulation to 40·1 and 35·5IU/L, respectively, with a concomitant low serum oestradiol concentration (7·4pg/ml, NR 10-400). Three months after rhGH treatment commencement, puberty spontaneously developed, progressing to Tanner stage 4 with a spontaneous 4-day menarche after 16 months on therapy. No further menstrual cycles occurred, with persisting low serum oestradiol concentrations and normal to high FSH and LH concentrations both at baseline and post-GnRH stimulation, thus necessitating hormone replacement. Patient S2 spontaneously commenced puberty at 11·6y, progressing to Tanner stage 4 at age 13y, but remaining amenorrhoeic up to her last evaluation (age 15·7y), with a baseline oestradiol of 9·8pg/ml, FSH 44·2IU/L and LH 12IU/L, peaking post-GnRH stimulation to 63·1 and 49·5U/L, respectively, and also requiring oestrogen replacement. Patient S3 commenced spontaneous puberty at age 13y, progressing to Tanner 4 over a 12-month period, with spontaneous menarche at 13·9y and later regular menstrual cycles with mildly elevated FSH and LH concentrations up to her last evaluation at age 14·1y. Normal sized/small ovaries but with sparse or absent follicles were found in all three siblings by ultrasonography. Russian patient R1 also had an elevated FSH concentration in the face of a normal LH at age 3y. Pelvic ultrasonography revealed a small uterus with no visible ovaries (Table-2).

Five male patients in this cohort developed normal puberty progressing to Tanner stage 5 with normal gonadotropin and testosterone concentrations. The youngest male (age 2y) also had a normal mini-puberty (Table-S2). Patient M1 fathered a healthy child at age 30y.

The details of clinical and laboratory data are given in Tables 1, 2 and S2 and Appendix.

#### Additional findings/neuropathy

In pedigrees 1-5, sitting height and upper-lower segment ratios were slightly increased (+2·2 to +2·6 SDS) and physical examination was otherwise normal except for a slightly high arched palate in all patients. On follow-up, patient T7 developed lower leg pain and bilateral pes cavus at age 22y. He was diagnosed with polyneuropathy based on nerve conduction studies with an elevated serum creatine phosphokinase (CPK) concentration (475U/L, NR 35-195).

In pedigree 6, patient S2 presented with reduced deep tendon reflexes and elevated CPK concentrations and was diagnosed with mixed motor neuropathy at age 16y. Electromyogram revealed moderately severe symmetric demyelination in both upper and lower limbs.

In pedigree 7, at age 5y, patient M1 became ataxic with frequent falls; increased CPK concentrations led to a diagnosis of myopathy. Two muscle biopsies were inconclusive, with degenerative nervus suralis changes on EMG.

In pedigree 9, patient I1 manifested minor dysmorphisms including prominent forehead, deep set eyes, prognathism, dental malocclusion, thin upper lip and clinodactyly. He had mild global developmental delay improving with time. During early childhood he experienced a few generalized motor seizures responsive to valproate. He also had a steppage gait and reduced deep tendon reflexes with mildly increased CPK, suggesting an underlying neuropathy. Nerve conduction studies showed electrophysiological findings consistent with a length-dependent axonal neuropathy more significant in the lower limbs. An NGS panel including more than 150 genes related to various neuropathies revealed no pathogenic variants.

### Molecular assessment

Variants identified in *RNPC3* in the patients are shown in Fig.1B. The localisation of the mutated residues within the hydrophobic core of the C-terminal RNA recognition domain of RNPC3 suggest that pathogenic variants could impair protein function and/or structure (Fig.1C).

In pedigree 1-5, in six patients (5F,1M), NGS identified a novel homozygous missense variant c.1449A>T, p.L483F in *RNPC3*. This variant was not reported in any public database, including 1000 Genomes, dbSNP, EVS or the gnomAD (v2.1.1) Browser (accessed on 05 April 2021). Additionally, a homozygous missense variant in *PRMT6*, NM_018137.2:c.1049C>G, (p.P350R), was identified in the same individuals that possessed the homozygous *RNPC3* (p.L483F) variants. Both genes are located in close proximity to each other (~4 Mb, Fig.S3) and due to the large region of homozygosity spanning this locus, variants in both genes have co-segregated in these families. Both variants were confirmed by Sanger sequencing. Affected patients were homozygous for both variants; the parents were heterozygous, and unaffected siblings were either heterozygous or homozygous wild-type. In pedigrees 6-9, Sanger sequencing confirmed compound heterozygosity for two identified variants, one being the missense *RNPC3* c.1420C>A, p.P474T variant, in each pedigree. The novel variants in pedigrees 6-9 were predicted to be deleterious using different prediction programs detailed in the Appendix.

Conservation of the mutated amino acids, RNPC3 (p.L483F), (p.P474T), (p.R205X), (p.R502X) and (p.P474LfsX10) across multiple species is shown in Fig.1D. The novel PRMT6 (p.P350R) missense substitution is also conserved across four species and is not present in homozygous state in control databases (1000 Genomes, EVS or the gnomAD Browser).

### Developmental expression pattern of *RNPC3*

**Figure 3:**
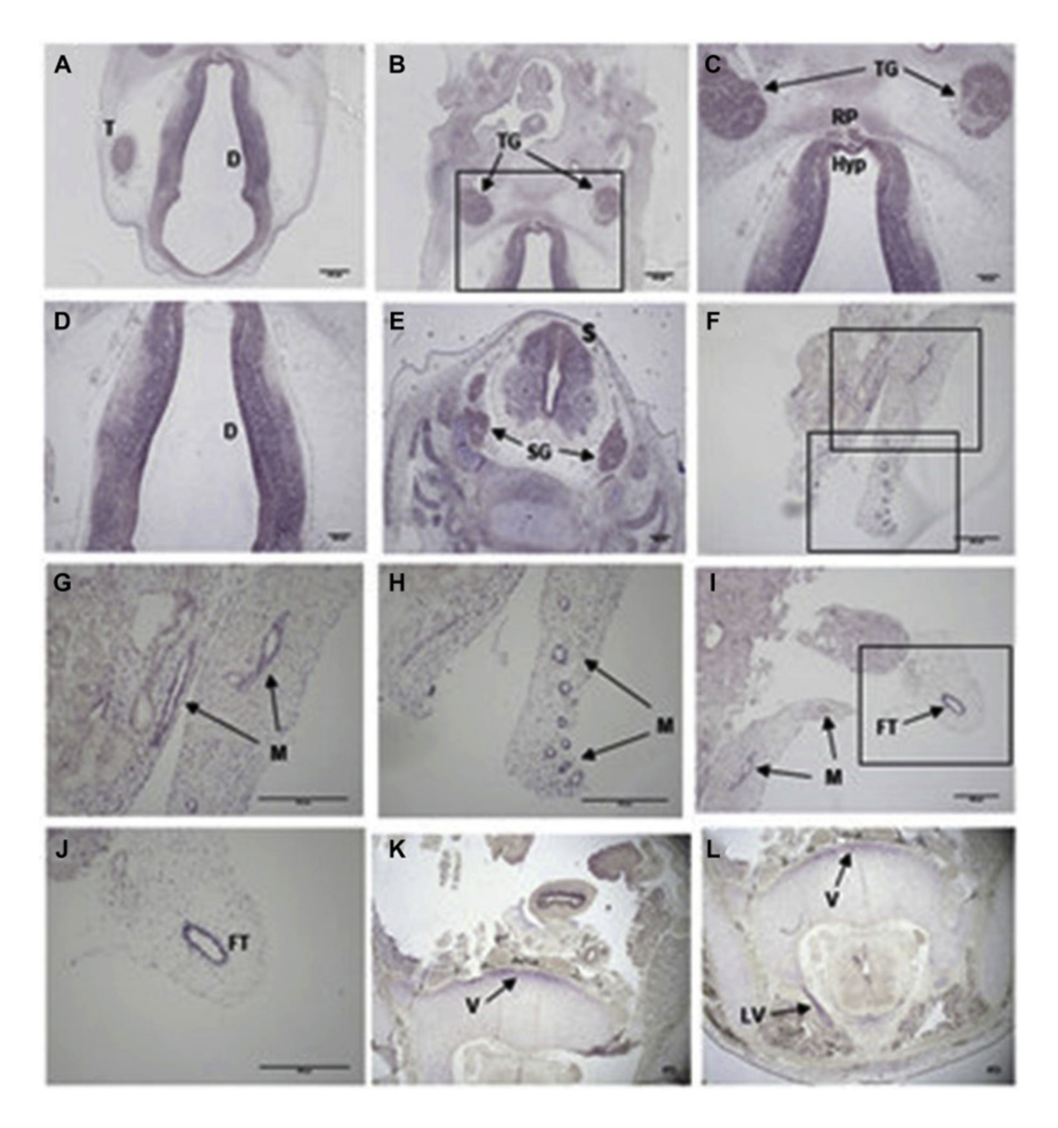
*RNPC3* expression in the developing hypothalamic-pituitary axis during human embryogenesis. *In situ* hybridization using the antisense probe against the human *RNPC3* mRNA transcript (*hRNPC3*) on human sections from different developmental stages during embryogenesis. (A-D) CS19: high *hRNPC3* expression is seen in the telencephalon, diencephalon, trigeminal ganglia and Rathke’s pouch. (E) CS19: expression can be seen in the spinal cord and spinal ganglia. (F-I) 9 post conception week (pcw): *hRNPC3* mRNA transcripts are present in the mesonephros; the ducts that will develop into the kidney, indicated by the labelled arrows. The boxes in F correspond to G and H respectively. (I-J) 9 pcw: expression is noted in the presumptive developing fallopian tube. The box in I corresponds to J. (K-L) 10 pcw: hRNPC3 expression is seen in the vertebrae and lamina of the vertebrae, indicated by the labelled arrows. T, telencephalon; D, diencephalon; TG, trigeminal ganglia; RP, Rathke’s \pouch; Hyp, hypothalamus; S, spinal cord; SG, spinal ganglia, M, mesonephros; FT, fallopian tube; V, vertebrae; LV, lamina of the vertebrae.

The Human Protein Atlas states that *RNPC3* is variably expressed throughout the human body including the pituitary gland, hypothalamus, and ovary. ISH performed on human and mouse embryonic tissue in this study revealed *RNPC3/Rnpc3* expression in the telencephalon, diencephalon, trigeminal ganglia, spinal cord and spinal ganglia, the hypothalamus and Rathke’s pouch (Fig.3A-E, Fig.S4). Additionally, in embryonic sections of the pelvic area, *RNPC3* was expressed in the mesonephros (the preliminary kidney ducts), fallopian tube, and vertebrae (Fig.3F-L). Furthermore, *Rnpc3* was expressed in the ovaries and hypothalamus, predominantly in ventromedial, dorsomedial and arcuate nuclei, in adult mice. *Prmt6* was also shown to be expressed in the embryonic hypothalamus, pituitary and ovary, as well as in the adult hypothalamus and ovary, in mice (Fig.S4).

### *Rnpc3* pathogenic variants are associated with GHD in female, but not male, mice

Homozygous *Rnpc3 ^p.L483F/p.L483F^* mice generated by Crispr/Cas9 technology were born in normal Mendelian ratios. Growth curves for the mutant male mice were normal. *Rnpc3* mutant female mice (n=5) were slightly lighter compared to wild-type females (n=5), although this difference was not statistically significant (p=0·09) (Fig.S5). Radio-immuno assays (RIA) performed on female pituitaries comparing homozygous mutants and wild-type littermate controls showed a decrease in GH contents in both sgRNA1 and 5 strains, reaching statistical significance only in sgRNA1 (64.28±4.6 SEM in controls, n=5, versus 43·4±3·5 SEM in mutants, n=4, p=0·016) (Fig.2). There was no significant difference for the male GH contents in the sgRNA1 strain (66·7±9·0 SEM in controls, n=7, versus 61·5±7·3 SEM in mutants, n=7). There was no significant difference in LH, FSH or PRL levels in homozygote mutant sgRNA1 females (XX, n=4) versus wild-type littermates (XX, n=5) (LH:0·275μg±0·04 SEM vs 0·385μg/pituitary±0·02 SEM, p=0·6, FSH:55·44ng±18·09 SEM vs 41·21ng±10·63 SEM, p=0·9, PRL:7·34μg±1·45 SEM vs 9·52μg±0·52 SEM, p=0·4). Histological examination of sexually mature female ovaries revealed no abnormality.

Homozygous *Prmt6 ^p.P350R/p.P350R^* mice were also generated by Crispr/Cas9. Growth curves for homozygous *Prmt6 ^p.P350R/p.P350R^* animals were normal (data not shown). Therefore, we only performed RIAs for GH in one of the two lines in females. There was no significant difference in pituitary GH content (GH 72·9±27·1 SEM for wild-type, n=2, and 74·4±6·1 SEM for mutants, n=3) (Fig.S1).

For fertility studies, eight-week-old females were mated with wild-type males for 10 weeks. There was no obvious difference in the average number of litters between *Rnpc3 ^p.L483F/p.L483F^* (3·8±0·4 SD, n=5) and wild-type (3·0±0·8 SD, n=4) females. The average number of pups/litter was also comparable (8±1SD pups/litter for *Rnpc3 ^p.L483F/p.L483F^* females, 7·2±2·6 SD for controls). While these results suggest that fertility is initially normal in female *Rnpc3* mutants, it would be interesting to test fertility over a longer period.

### Re-analysis of the RNAseq Data for POI and peripheral neuropathy phenotypes

To test the splicing efficiency of U12-type introns in candidate genes which might be involved in two novel phenotypes, POI and/or peripheral neuropathy, we re-analyzed the RNAseq data obtained previously from total RNA extracted from mononuclear blood cells of two patients (S1 and S2) and four controls.^14^ We listed the U12-type intron containing genes which were documented or candidates to be involved in POI and/or peripheral neuropathy. The splicing efficiency and intron retention of U12-type introns were quantified with respect to U2-type introns per gene, normalized by gene expression. Although several candidate genes did not have sufficient expression levels in blood cells to detect abnormalities, abnormal processing of U12 introns in patient cells were striking for *HARS and GARS* (Table-S3), reported to be involved in autosomal dominant neuropathies.^16,17^

## Discussion

Using NGS, we identified a series of biallelic pathogenic variants in *RNPC3* in 15 patients from nine families with severe GHD. Most affected females had evidence of POI (n=8) while males (n=6) had normal pubertal development and gonadotropin concentrations, with fertility documented in the eldest male patient.

Long thought to be a default pathway occurring in the absence of SRY, ovarian development is now known to be an active process involving several regulatory genes.^18^ POI, with gonadal dysgenesis at the most severe end of the phenotypic spectrum, can be caused by genetic factors. To date, variants in over 50 genes have been associated with POI, overall accounting for a small proportion of patients; the aetiology remains to be elucidated in most cases.^2^ These gene variants can affect various processes such as gonadal development, DNA replication/meiosis and repair, hormonal signaling, immune function and metabolism.^19^ Here, we report that pathogenic variants in *RNPC3* encoding U11/U12-65K, a specific protein component of the minor spliceosome, are associated with POI through a pathway that is distinct from the previously reported mechanisms.

The U12-dependent spliceosome includes four unique small nuclear RNAs (snRNAs) designated as U11, U12, U4atac, and U6atac, and seven unique proteins, all found on the U11/U12 di-snRNP, which recognizes the first intron.^20,21^ Compound heterozygous pathogenic variants in *RNPC3* were initially reported to cause IGHD.^14^ Both variants, namely missense (p.P474T) and nonsense/truncating (p.R502X), were shown to reduce formation of the U12-type intron recognition complex, leading to splicing defects in a subset of minor introns.^14,22^ A more recent study described compound heterozygosity for two variants in *RNPC3* in three siblings from a Caribbean pedigree, associated with GH, TSH and prolactin deficiencies, as well as learning difficulties and congenital cataracts. Pubertal delay due to apparent hypogonadotrophic hypogonadism was described in two of the siblings, but biochemical data showing abnormal gonadotropin secretion and details of pubertal development and any treatment required to progress puberty were not provided.^23^ During the submission process of the current paper, another patient with severe primordial microcephalic dwarfism and intellectual disability was reported to be carrying compound heterozygous variants in *RNPC3*. She was reported to be prepubertal at age 11y; GHD and prolactin deficiency were documented but biochemical data of gonadotropin concentrations were not provided.^24^

Since the first three publications of *RNPC3* variants in cases with GHD,^14,23,24^ we have identified 12 more patients, four compound heterozygotes for p.P474T together with a second disruptive change and eight homozygotes with p.L483F variant. The first three Spanish siblings reported^14^ presented with severe isolated GHD at ages 14y, 7·6y and 5·5y, respectively. The eldest patient developed spontaneous puberty at age 15·7y, shortly after commencement of rhGH treatment, and had menarche at age 16·7y. However, no further menstruation occurred, and the diagnosis of POI was later established. The other two siblings entered puberty spontaneously at ages 11·6y and 13y, respectively. Nevertheless, their gonadotropin concentrations also increased progressively and ultrasonographic investigations revealed normal/small ovaries with sparse or absent follicles. Contrastingly, proband 1 in the Turkish pedigree presented with POI at age 14y. The other four female patients harbouring the same variant also showed evidence of ovarian insufficiency. Although spontaneous puberty started at age 11·9y and 11·8y in patients T2 and T3, the ovaries have been undetectable or very small with no follicles in all Turkish patients throughout the follow-up period, and hypergonadotropic hypogonadism developed eventually. The Russian patient also had elevated FSH and pubertal LH concentrations at age 3y, consistent with the findings in the Turkish girls during the prepubertal period, although a definitive diagnosis of POI cannot be made as yet.

U12-type introns are particularly present in genes related to ‘information processing functions’, such as DNA replication and repair, transcription, RNA processing, and translation.^25^ Impaired minor class splicing generally leads to intron retention and, less frequently, exon skipping and alternative splicing.^26^ These perturbations often result in frame-shifts, creation of premature stop codons and reduced expression and function of affected genes.^27,28^

We hypothesized that splicing defects in genes involved in ovarian development or function may be implicated in POI in our female patients with *RNPC3* variants. Therefore, we searched for all the U12-type intron containing genes possibly or proven to be associated with POI and/or ovarian development, by examining the most updated human and animal studies in the literature. Several candidate genes were shortlisted, including *DIAPH2, EXO1, SPO11, NUP107 and PTEN*. Given the tissue-specific importance of Nup107 for ovarian development, and female-specific sterility in knockdown animals,^29,30^ *NUP107* seems to be a strong candidate to possibly explain the POI in our patients, requiring further study. In patients S1 and S2, re-analysis of RNAseq revealed increased retention of U12-type introns in *MCMBP,* encoding a component of the mini-chromosome maintenance (MCM) complex which acts as a regulator of DNA replication (Table-S3). Therefore, this gene might also be a good candidate for this phenotype, although it has not yet been reported to be associated with POI.

Of the approximately 50 genes in which variants clearly cause POI in humans, only half have relevant mouse models.^19^ In the current study, in the mouse model harbouring the homozygous p.L483F variant, although there was female-specific GHD, the ovaries were grossly normal and fertility was not impaired in young mice. Eight-week-old *Rnpc3 ^L483F/L483F^* female mice that were mated with males for 10 weeks appeared to have normal ovaries. It should, however, be noted that subfertility was reported in 12-month-old *Nup107^R356C^* mutant mice, with normal ovarian structure and size at 6 months of age.^30^ Hence, these mice may be too young to manifest an ovarian phenotype. Another possible reason for the lack of an ovarian phenotype in mice may lie in the difference between human and murine reproductive phenotypes, as observed in Turner syndrome, where humans are mostly infertile while mice are fertile but have a reduced oocyte pool and reproductive lifespan.^31^

In this cohort, almost all patients had anterior pituitary hypoplasia with a normal stalk and a eutopic posterior pituitary of normal hyperintensity. The expression studies performed on human embryonic sections in this study further suggest an important role for *RNPC3* during HP development, with strong expression throughout the diencephalon, hypothalamus and Rathke’s pouch (Fig.3A-D).

Assessment of pituitary function revealed that the corticotrophic axis and gonadotropin production and secretion were intact in all patients. Overt central hypothyroidism was diagnosed in two patients, while mild thyroid dysfunction was observed in a further three patients. However, interestingly, PRL concentrations were strikingly low in most affected patients. The phenotype of CPHD involving somatotrophs, thyrotrophs and lactotrophs is consistent with the findings in the Caribbean pedigree,^23^ although the TSH deficiency appeared to be more severe in the latter pedigree.

The growth response to rhGH therapy was excellent in all patients with *RNPC3* variants. In 10 patients who were treated with rhGH regularly, height changed between 1·1 and 7·5 SDS after 1-9 years. Growth almost ceased in one female patient (T4) during interruption of therapy for 21 months at around 10 years of age, with growth acceleration upon recommencing rhGH. Contrastingly, two of the males displayed normal growth off GH treatment, despite manifesting severe biochemical GHD. This relatively milder GHD phenotype in male patients may be consistent with the lack of a phenotype observed in the *Rnpc3* mutant male mice we generated. This may reflect a difference in the extent of GHD between males and females, as was also observed with gonadal susceptibility in humans.

Furthermore, a history of mild developmental delay and/or ataxia or neuropathic/myopathic symptoms led to the diagnosis of a motor neuropathy in patients M1, I1, T7 and S2 based on abnormal nerve conduction studies and elevated CPK concentrations. Recently, a variant in the U12 snRNA gene (RNU12), probably affecting the binding site for the 65K protein, has been associated with early-onset cerebellar ataxia (EOCA). RNAseq analyses indicated increased U12-type intron retention along with U12 small nuclear ribonucleoprotein upregulation.^32^ There is mounting evidence suggesting that the minor spliceosome contributes to the pathophysiology underlying neurodegenerative disorders such as spinal muscular atrophy.^33,34^ Taking into account that patients with pathogenic variants in *RNPC3* from four different pedigrees had similar neurological findings, we speculate that splicing defects in some U12-type introns in neurons may contribute to this phenotype in our patients. Re-analysis of the RNAseq data of our patients S1 and S2 revealed significantly defective splicing of *HARS1* and *GARS1* in patients compared to controls (Table-S3). Further functional studies are needed to investigate any possible mechanistic relationship of this phenotype to *RNPC3* variants. Furthermore, the occurrence of rapidly progressive glomerulonephritis in patient M2 from pedigree 7 remains to be understood in terms of causality or coexistence. Conceivably, the *RNPC3* expression seen in the mesonephros of the human embryonic kidney may explain this phenotype. Likewise, the strong expression seen in the spinal cord and spinal ganglia in human embryonic sections (Fig.3E), and additionally in the vertebrae in pelvic sections (Fig.3K-L) of human embryos, supports a contributory role of RNPC3 towards neurological phenotypes.

In summary, two of three previously reported Spanish patients with *RNPC3* variants developed ovarian insufficiency on follow-up, with elevated gonadotropins and small ovaries in the third. Thereafter, six other girls from different families (five Turkish and one of Russian origin) have been identified with the same phenotype of severe GHD associated with proven/possible POI. Causality is supported by the identification of different *RNPC3* variants in patients with a similar phenotype from different ethnic origins.

To conclude, we have further expanded the phenotypic spectrum associated with *RNPC3* variants. A novel homozygous missense pathogenic variant (p.L483F) in *RNPC3* was associated with GHD, hypoprolactinaemia, variable TSH deficiency and anterior pituitary hypoplasia in humans. The majority of female patients also present with POI, while males have normal pubertal development and gonadal function including fertility. Data in humans are partially supported by murine studies which show female-restricted GHD, but normal gonadal function, and by *RNPC3* expression in the HP region of the human brain, further supporting a role for this gene in HP development. Currently, we cannot explain the differences between the males and females, but this could be the foundation for future studies. Our findings suggest novel insights into the role of RNPC3 in ovary-specific gonadal function and emphasize a critical role of the minor spliceosome in the processing of genes required for pituitary and ovarian development and function.

## Supplementary Material

Supplementary Materials

## Figures and Tables

**Table 1:** Auxological and hormonal parameters and anterior pituitary height on MRI of the patients at diagnosis and the growth response after rhGH treatment

Patient ID	Sex	Age (y)	Bone Age(y)	Height(SDS)	Weight(SDS)	BMI(SDS)	Growth Velocity (cm/y)	Growth Velocity (SDS)	GH (ng/dL) Peak After Clonidine or Arginine	IGF-1 (ng/mL)	IGFBP-3 (mg/L)	Prolactin (ng/mL)	Cortisol (ug/dL)	TSH (U/L)	Free T4(ng/dL)(Normal Range)	Anterior Pituitary Height on MRI(*n* for age)	First Year Growth After rhGH (cm/year)	Final Height(SDS)	Mid-Parental Height (SDS)	Height SDS Change^a^
T1	F	8.2	2.6	−8.9	−6.1	0.3	2.5	−2.6	<0.01	0.01	1.7	1.8	14.1	2	0.7 (0.7-1.7)	2.5 (4.4 ± 0.9)	6	−2.7	−1.6	6.8
T2	F	5.1	2	−8.7	−7.3	0.0	2.4	−3.1	0.01	0.01	<0.5	1.7	12.3	3	0.8 (0.8-2.0)	1.5 (4.9 ± 0.9)	17.5	−2.2	−1.5	6.7
T3	F	3	1	−6.5	−4.7	0.1	4.0	−2.3	<0.01	0.01	<0.5	1.2	10.8	7.5^b^	0.8 (0.8-2.0)	4 (4.2 ± 0.6)	17	GC	−2.1	5.1
T4	F	2.2	0.7	−4.4	−1.5	2.0	4.5	−2.5	0.40	<10	6.8	9.1	13.3	10.6^b^	1.0 (0.8-2.0)	NA	15.1	GC	0.9	3.7
T5	F	5.5	2	−6.5	−4.5	0.3	4.0	−2.1	0.12	<25	<0.5	3.2	16.1	2	0.9 (0.6-1.1)	3 (4.2 ± 1.0)	12.6	NA	−1.6	NA
T6	M	9.7	5	−7.2	−3.5	1.3	4.4	−0.9	<0.01	2.4	NA	1.1	14.0	3.1	0.7 (0.8-1.7)	2 (4.2 ± 1.2)	7.5	−4.9	−1.4	1.6
T7	M	6.6	2	−8.5	−8.8	−0.5	1	−4.2	0.01	<0.01	NA	NA	15.2	6.7^b^	1.0 (0.6-1.1)	3 (5.0 ± 0.7)	11.5	−1.8	−1.4	7.5
T8	M	1.1	0.6	−4.2	−2.0	1.1	6.7	−1.6	0.06	15.0	<0.5	2.8	17.4	1.7	0.8 (0.9-1.7)	2.5 (4.1 ± 0.7)	14.1	GC	−2.0	1.1
S1	F	14.0	10	−9.6	−2.6	2.8	1.7	−2.1	<0.5	36	0.8	0.7	20.4	4.2	0.9 (0.6-1.4)	3.5 (6.1 ± 1.0)	12.8	−1.8	−0.9	4.0
S2	F	7.6	4.5	−5.0	−1.8	0.1	1.5	−3.5	<0.5	18	<0.5	2.1	14.2	4.5	0.9 (0.6-1.4)	2.5 (4.6 ± 1.3)	14.2	0.9	−0.9	4.0
S3	F	5.5	2	−6.7	−3.5	−1.5	1.7	−4.0	<0.5	21	<0.5	1.8	20.0	3.2	0.9 (0.6-1.4)	2.5 (3.8 ± 1.0)	14.6	GC	−0.9	5.0
M1	M	2.3	0.5	−5.9	−3.1	1.1	2.5	−2.6	<0.3	NA	NA	NA	12.5	3.2	12.0^c^ (4.5-12.5)	3.5 (4.2 ± 0.6)	22	0.0	−1.7	5.9
M2	M	1.8	0.8	−5.0	−1.9	1.3	3.0	−2.5	<0.3	NA	NA	NA	13.0	4.2	11.0^c^ (4.5-12.5)	2.5 (4.1 ± 0.6)	13	−2.8	−1.7	2.2
R1	F	3	1.7	−4.1	−2.3	1.3	4.8	−1.8	0.6	10.9	—	18.3	22.9	1.8	1.3 (1.1-2.0)	3.0 (3.4 ± 1.0)	13.3	GC	−0.1	
I1	M	3.2	1.5	−6.5	−3.7	2.0	—	—	<0.05	14	—	<0.5	13.4	0.9	0.6 (0.8-1.9)	4 (5.0 ± 0.7)	15.7	−1.1	−0.8	4.5

Conversion factors from conventional units to SI units: GH (ng/mL) to μg/L, multiply by 1. IGF-1 (ng/mL) to nmol/L, multiply by 0.131. Prolactin (ng/mL) to μg/L, multiply by 1. Free T4 (ng/dL) to pmol/L, multiply by 12.871. Total T4 (μg/dL) to nmol/L, multiply by 12.871. Cortisol (μg/dL) to nmol/L, multiply by 27.588. *F*, female; *GC*, growth continues; *GH*, growth hormone; *M*, male; *MRI*, magnetic resonance imaging; *NA*, not available; *rhGH*, recombinant human growth hormone; *T4*, thyroxine; *TSH*, thyroid-stimulating hormone; *SDS*, SD, score.

a Height SDS change from the onset of rhGH to the latest evaluation.

b Values above the upper limit of TSH.

c Total T4 (μg/dL). Prolactin normal range, 3.5 to 25 ng/mL. Cortisol normal range, 5 to 25 μg/dL.

**Table 2:** Pubertal status, serum gonadotropin concentrations and ultrasonographic findings of uterus and ovaries at the diagnosis and on follow-up in the female patients

Patient ID	T1			T2			T3			T4			T5	SI			S2		S3			R1
Age (y)	8	14	21	5.1	13	17.5	3.1	7.1	11.5	3.8	7.4	10.9	5.9	15.7	18.5	19.5	15.0	16	12.9	13.4	13.9	3
Tanner stage	1	2	5	1	2	5	1	1	2	1	1	1	1	2	5	5	4	5	1	3	4	1
FSH (U/L)	24.8	86.6	35.9	7.8	38.3	7.5	20.9	8.7	30.1	55.2	12.6	39.8	16.3	30.3	29.2	2.7	52.8	17.6	11.1	18.1	19.1	37.4
LH (U/L)	0.7	32.1	43.8	0.1	28.7	6.3	0.9	0.07	5.9	0.6	0.0	2.1	0.1	6.7	12.9	1.9	20.2	11.4	0.3	5.8	3.3	1.7
E2 (pg/mL) (USG)	<5	15	NA	<5	<5	34	NA	NA	<5	NA	<5	<5	<5	7.4	12	10.2	11	68	7.5	32	35	
Uterus volume (mL)	0.2	0.6	5	0.5	4	4	0.3	0.3	0.4	0.3	0.3	0.3	0.2	1.4	5.9	19.3	17	39.2	NA	NA	29.2	0.4
Ovary-right																						
Volume (mL)	NV	0.06	2.2	NV	NV	NV	NV	NV	0.3	NV	NV	0.2	NV	1.7	0.5	0.5	3.3	l.6	NA	NA	2.5	NV
Follicle number	0	0	1	0	0	0	0	0	0	0	0	0	0	0	0	0	1	3	NA	NA	4	0
Follicle size (max mm)	-	-	6	-	-	-	-	-	-	-	-	-	-	-	-	-	4.5	2	NA	NA	3	-
Ovary-left																						
Volume (mL)	NV	0.07	1.9	NV	NV	NV	NV	NV	0.3	NV	NV	0.3	NV	NV	0.4	0.4	NV	1.9	NA	NA	2.3	NV
Follicle number	0	0	0	0	0	0	0	0	0	0	0	0	0	0	0	0	0	6	NA	NA	3	0
Follicle size (max mm)	-	-	-	-	-	-	-	-	-	-	-	-	-	-	-	-	-	2	NA	NA	2	-
E2 replacement	-	-	+	-	-	+	-	-	-	-	-	-	-	+	-	+	-	+	-	-	-	-
Age of spontaneous thelarche (y)		14			11.9			11.8			-		NA		15.7		11.6		13		-
Age at initiation of E2 therapy(y)		17			16.5			12.5			12.5		NA		19.2			15.0		-		-

For conversion of E2 (pg/mL) measurements to SI unit pmol/L, multiply by 3.671. *E*, Estradiol; *FSH*, follicular stimulating hormone; *ID*, Identification; *LH*, luteinizing hormone; *NA*, not available; *NV*, not visible; *R*, Russion; *S*, Spanish; *T*, Turkish; *USG*, ultrasonography.

## Data Availability

The variants described in this publication have been submitted to ClinVar (Submission ID:SUB9429499).
