# Supplementary material for: Pathogenic Variants in RNPC3 are Associated with Hypopituitarism and Primary Ovarian Insufficiency
Source: Genet Med. Author manuscript; Available in PMC 2022 Feb 16. (PMC7612377; doi:10.1016/j.gim.2021.09.019)
Supplement: Supplementary Materials [file EMS142040-supplement-Supplementary_Materials.pdf]

SUPPLEMENTAL APPENDIX

Supplemental Methods

1. Next generation sequencing and data analysis

Whole-exome capture was performed using the following captures i) Pedigrees 1-5: SureSelect version 4 kit (Agilent, Santa Clara, CA), ii) Pedigree 7: SeqCap Roche Human Exome V3 capture system (Roche NimbleGen, Madison, WI, USA), iii) Pedigree 8: GenSeq™ DNA Library kit v1.1 (Anpro, Moscow, Russian Federation) and a custom Ion Ampliseq™ panel (Ion Torrent, Thermo Scientific, USA) targeting 19 genes associated with growth hormone deficiency (*ACAN, ARNT2, GH1, GHRH, GHRHR, GHSR, GLI2, HESX1, IGSF1, LHX3, LHX4, OTX2, PAX6, POU1F1, PROP1, RNPC3, SHH, SOX2, SOX3*) and iv) Pedigree 9: SureSelect Clinical research exome 54Mb (Agilent Technologies) and exonic sequences were captured with the xGene Exome Research Panel (IDT). Illumina platform (Illumina, San Diego, CA, USA) was used for sequencing.

Reads were aligned to the human reference genome (build GRGh37/hg19) using Burrows-Wheeler Aligner (BWA) algorithm[1] or Novoalign (www.novocraft.com). Bioinformatics analysis was carried out using Genome Analysis Tool Kit (GATK; Broad Institute, Cambridge, MA, USA).[2] Variants were annotated using Ingenuity Variant Analysis software (version 4.1.20160526; www.ingenuity.com/variants) in Pedigrees 1-5, CLC Genomics Workbench version 12.0 (CLCBIO, Aarhus, Denmark) in pedigree 9 and by ANNOVAR[3] in Pedigrees 7 and 8.

As whole exome variants were processed at different times and at several institutions/countries, the filtering parameters applied varied. In Pedigrees 1-5, the following variants were kept: quality score ≥20 and read depth ≥10, population frequency ≤1% (1000 genomes, NHLBI Exomes, ExAC), exonic changes other than synonymous, intronic splice sites (affecting first 7 bases of an intron), synonymous changes that are predicted to affect splicing as determined by MaxEntScan. In Pedigree 7, the following variants were kept: quality score ≥20 and coverage ≥4X, located outside of segmental duplications and simple repeats, variants with allele frequency ≤1% in the 1000 Genomes or NHLBI Exomes, non-synonymous variants with Polyphen2[4] scores >0.8 and SIFT[5] scores ≤ 0.05, splice-site (±15 nt), nonsense, non-stop, and small in-frame or frame-shift indels. In Pedigree 8, quality score ≥20 and coverage ≥10X, exonic variants, intronic variants within 10 bases from exon-intron boundaries, minor allele frequency ≤0.1% (gnomAD) were kept. In Pedigree 9, non-synonymous exonic changes and invariant splicing variants with a minor allele frequency ≤0.1% in the Genome Aggregation Database (gnomAD) were kept.

In all pedigrees, variants were assessed for segregations under the following inheritance models: X-linked, *de novo* and autosomal recessive (homozygous and compound heterozygous).

Methodology of the whole exome sequencing performed in Pedigree 6 was reported previously.[6]

2. Sanger sequencing confirmation

The variants identified by WES in each pedigree were confirmed by Sanger sequencing. Genomic DNA was isolated from leukocytes of peripheral blood by using standard protocols in each individual. The regions of interest were amplified by PCR using primers depicted in Table S1 and sequenced using BigDye Terminator V.3.1. Reference sequences were obtained from NCBI (reference sequence: NM_017619). The PCR amplification conditions for each reaction are available upon request.

3. Predicting the pathogenicity of novel variants in RNPC3

We predict that p.Pro474Leufs*10 is pathogenic as it at the same codon as a known pathogenic variant. The proline amino acid has a large side chain and therefore both, the known pathogenic change to Threonine (p.Pro474Thr), and our Leucine change will have a fundamental deleterious effect on the protein. Based on Deciphering Developmental Disorders (DDD) study (Decipher), any truncating changes beyond amino acid 481 in RNPC3 are predicted to be subjected to NMD (nonsense-mediated decay). Therefore, the variant p.Pro474Leufs*10 is predicted to be subjected to NMD or truncated protein missing 44 amino acids including an RNA-binding domain.

The figure below has pathogenic variants from the paper in red, exons in grey, coding sequence in yellow and protein domains in blue (made using geneious prime 2021.1.1):

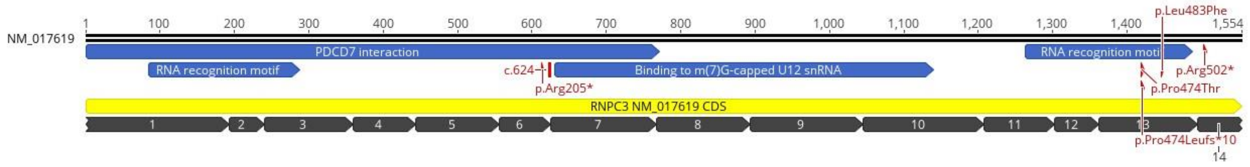

With respect to the p.Arg205* variant, there is precedence for nonsense variants to be pathogenic in this gene and the gene has a pLoF score of 0.19 which means that it is highly constrained and does not tolerate loss of function variants. The variant at position c.613C>T in intron 6/14 creates a premature stop codon at amino acid 205; the RNA transcript is likely to be subjected to nonsense-mediated decay; any transcripts escaping nonsense-mediated decay would encode for a severely truncated protein missing 312 amino acids and several functional domains including part of the PDCD7 binding domain and the entire m(7)G-capped U12 snRNA binding domain. Total length of the RNPC3 protein is 517 amino acids.

As for the splice site variant c.624+1G>T, it affects the highly conserved residue in the canonical splice site and is thus predicted to cause splicing defects with high confidence. The four in silico algorithms (SSF, MaxEnt, NNSplice and HSF) unambiguously agree that variant RNPC3 c.624+1G>T is predicted to cause loss of the canonical donor site and is likely to result in an in-frame exon skipping event of at least exon 6 of the NM_017619.3 transcript or likely more (Alamut Visual version 2.9.0, Interactive Biosoftware, Rouen, France). We used the Tayoun ACMG PVS1 guidelines to predict the effect of this on the reading frame[7]. RNA studies are needed to confirm the exact effect of c.624+1 on splicing.

4. *In situ* hybridization studies

Murine expression was analysed in C57Bl6 samples as previously described[8] using probes synthesized from cloned PCR fragments spanning nucleotides 17 to 820 for *Rnpc3* and 524 to 1479 for *Prmt6* of the corresponding coding sequences. Human brain and pelvic sections at different stages of embryonic development were provided by the Joint MRC/Wellcome Trust (grant# MR/R006237/1) Human Developmental Biology Resource (HDBR) (http://hdbr.org). A purified pCMV-SPORT6 vector containing full length human wild-type *RNPC3* cDNA (IMAGE ID: 3873751) (Source Bioscience) was cut to make antisense and control sense digoxigenin-labelled RNA probes respectively. The *in situ* hybridization used to analyse human embryonic gene expression was performed as previously described.[9]

5. Mouse studies

We used the Crispr/Cas9 technology to generate mice carrying the p.Leu483Phe variant in the conserved RRM2 domain of murine *Rnpc3*. We also generated mice harbouring the p.Pro350Arg variant in *Prmt6*. A plasmid encoding the Cas9 protein, a single stranded oligonucleotide containing the variant of interest,

and sgRNA were injected in mouse C57Bl6 zygotes, as described previously.[10] The single stranded template also contained two synonymous variants in the guide region to avoid re-cutting once the targeting event had occurred. Two sgRNA were designed and independently injected to produce two different mouse strains for each of the genes, to discriminate between the effects of the desired variant, which would be common to both guides, and potential off-target effects of the guides, which would be different between the two lines (Figure 2, Figure S2). Animal samples were grouped by litters which comprised both wild-type and mutant animals, allowing for randomization and appropriate littermate controls. To prevent subjective bias, samples were not identified by genotype when hormone level measurements were performed.

6. Growth curves in mouse studies

Weight measurements were taken blindly by animal technicians who were not involved in the study. Mouse weights were regressed against time (in weeks), segmented by genotype, within a linear mixed effect model where the random effect is set at the mice's individual variations using the following formula:

Weight ~ log(Week) + 1_(Genotype = Mutant) + 1_(Genotype = Mutant) x log(Week)

Week is log-transformed to account for the fact that weight gain is rapid at first, but then plateaus as mice finish maturing. Mutant genotype is handled as a binary variable.

7. Mouse fertility studies

Eight-week-old *Rnpc3 p.Leu483Phe/ p.Leu483Phe* and wild-type littermate females were bred to C57Bl6 wild-type males. Each pair was housed in an individual cage for ten weeks. Litters were recorded at birth and removed before weaning stage.

8. Hormonal evaluation in patients

Hormonal assays were performed in the local laboratories and evaluated according to the normal ranges for each assay.

In pedigrees 1-5, The serum oestradiol and testosterone concentrations were measured using the ELECSYS Estradiol III and Testosterone II assay (Roche Diagnostics GmbH, Mannheim, Germany) by Modular analytics E170 autoanalyser. These assays employ the electrochemiluminescence immunoassay (ECLIA) method and have been standardized against CRM 6004a via isotope dilution-gas chromatography/mass spectrometry (ID-GC/MS). The coefficient of variation (CV) of oestradiol and testosterone were 8.5 % and 4.7 %, respectively.

Measuring range for oestradiol is 5-3000 pg/mL (defined by the Limit of Detection and the maximum of the master curve). Values below the Limit of Detection are reported as < 5 pg/mL.

Measuring range for total testosterone is 0.025-15.0 ng/mL (defined by the Limit of Detection and the maximum of the master curve). Values below the Limit of Detection are reported as < 0.025 ng/mL. Values above the measuring range are reported as > 15.0 ng/mL.

In pedigree 6, serum oestradiol concentrations were measured by The ARCHITECT Estradiol assay (Abbott; Abbott Park, Chicago; IL, USA), a delayed one-step immunoassay using chemiluminescent microparticle immunoassay technology. The analytical sensitivity of the ARCHITECT Estradiol assay is around 10 pg/ml. Calibration range: 0 to 1000 pg/ml. The ARCHITECT estradiol assay was compared to

isotope dilution-gas chromatography/mass spectrometry and gave a correlation coefficient of 0.99, with a mean slope of 1.09.

In pedigree 7, serum total testosterone concentrations were measured by chemiluminescent assay (Access Testosterone assay in Access 2 Immunoassay Systems). Dynamic measurement range is 10-1600 ng/dl. Inter-assay variation is 4.22-7.08%.

In pedigree 9, Serum testosterone was measured by using the Immulite 2000 which is an automated, random-access immunoassay analyzer with a solid-phase washing process and a chemiluminescence detection system. The interassay precision (CV%) is 13.7 with a calibration range: 20–1600 ng/dL.

Testosterone measured by most automated and manual methods are reported within 20% of those measured by LC-MSMS and capable of distinguishing eugonadal from hypogonadal males[11].

The diagnosis of central hypothyroidism is based on low serum thyroid hormone concentrations in the face of low/normal or slightly elevated TSH concentrations.

Cortisol deficiency was ruled out with normal early morning serum cortisol and ACTH concentrations, which are checked regularly in the patients during follow-up visits to the clinic.

Primary hypogonadism was diagnosed based on medical history, physical examination along with increased serum gonadotropin concentrations for age and pubertal stage in the face of low/undetectable serum oestradiol concentrations and supporting ultrasonographic findings[12]. In older patients, POI was diagnosed based on ESHRE guidelines and established diagnostic criteria[13] which are raised gonadotropins (FSH >40IU/L) taken three months apart on two occasions.

FSH and LH concentrations were measured by immunochemiluminometric assays (ICMA). Normative reported ranges for FSH in females: Tanner I girls (ages 2–9): 1.0–4.2 IU/l; Tanner II (ages 9.2–13.7): 1.0–10.8 IU/l; Tanner III (ages 10.0–14.4): 1.5–12.8 IU/l; Tanner IV (ages 10.7–15.6): 1.5–11.7 IU/l, and Tanner V (ages 11.8–18.6): 1.0–9.2 IU/l. Normative ranges for LH in females: Tanner I girls (ages 2–9): 0.02–0.3 IU/l; Tanner II (ages 9.2–13.7): 0.02–4.7 IU/l; Tanner III (ages 10.0–14.4): 0.10–12.0 IU/l; Tanner IV–V (ages 10.7–18.6): 0.4–11.7 IU/l.

9. Pelvic imaging protocol

The pelvic ultrasonography (US) examinations were performed by dedicated paediatric sonographers using a transabdominal approach in all patients. Transabdominal images were obtained after night fasting with repleted bladder and the patient in a supine position. At the time of examination, the sonographer measured all adequately visualized pelvic organs, including left and right ovaries and uterus, in three dimensions (length, width and height in centimetres), endometrial thickness (double wall) in millimetres and additional measurements of any pertinent findings if present. Uterine and ovarian volumes were subsequently calculated using the prolate ellipsoid formula (volume=lengthxwidthxheightx0.523). All images were reviewed by a paediatric radiologist at the completion of imaging and a clinical interpretation, including verified pelvic organ measurements, was provided based on the images.

In pedigree 1-5, Siemens Acuson Antares Ultrasound system (Siemens AG, Muenchen, Germany) with convex US probes with a bandwidth 1-4 MHz were used depending on sonographer preference and patient body habitus. In pedigree 6, pelvic ultrasonography studies were performed with a high-end Samsung RS80A ultrasound device with Prestige technology (high-quality S-vision) using a CA1-7AS-vue transducer (Samsung Electronics 2014). In pedigree 8, the Toshiba Aplio 500 TUS-A500 device was used.

Pelvic organ measurements have been evaluated according to the normative data for children and adolescents.[12]

Table S1: Primers used for PCR amplification and Sanger sequencing of variants.

	Gene	Exon	Forward Primer	Reverse Primer
Pedigree 1-5	*RNPC3*	14	TAGGAGAATGGCGTGAACCT	GCCTTTGCTTCTTTACCTATCTTG
Pedigree 1-5	*PRMT6*	1	CCTCAACAACGGATACAGCG	CCTTTCTCCCCAGCTTCAGA
Pedigree 7	*RNPC3*	6	AAGAGATGGGCAGGTCTTCC	CCGGCTTTTACTCACTTAACAG
Pedigree 7	*RNPC3*	13	TGACGTGCCTATCAAATAGTGT	CTACTGCTAGAATGCCAATTGTG
Pedigree 8	*RNPC3*	5-6	TATCATTTGAGCAGTGTGTTTG	TCAGCCTCAATAATCCCATTC
Pedigree 8	*RNPC3*	13-14	CGTGCCTATCAAATAGTGTAG	CTGAATTACTACCTTGTCAAAC
Pedigree 9	*RNPC3*	13	TTTTAGTGCAGAACATGAAATATATT	CTACTGCTAGAATGCCAATTG

Supplemental Results

Table S2: Pubertal data showing normal pubertal development, and normal serum gonadotropin and testosterone concentrations for age in the male patients.

Patient ID	Age (years)	Pubic hair Tanner Stage	Testis volumes (ml)	FSH (IU/L)	LH (IU/L)	Total testosterone (ng/dl)
T6	16.5	5	20/20	2.4	2.2	255
T7	17	5	25/25	1.4	2.4	350
T8	0.7	1	2/2	1.7	1.8	<2.5
M1	30	5	25/25	1.9	2.2	348
M2	21	5	25/25	1.7	2.8	220
I1	22.1	5	25/25	3.4	6.7	390

Total testosterone NR: Tanner stage 1: <0.2-3.4 ng/dl, Tanner stage 5: 180-880 ng/dl. For conversion of total testosterone measurements from ng/dl to SI unit nmol/L, multiply by 0.0347.

Table S3: The list of U12-type intron containing genes with documented or possible relationship with POI and/or neuropathic diseases (according to OMIM) and U12-type splicing efficiency on RNAseq data from mononuclear blood cells of patient S1and S2.

Gene	Function/mechanism	Documented genes for POI	U12sr/U2sr<br>ca/co ratio	U12ret/FPKM<br>ca/co ratio
HARS2	Histidyl tRNA synthetase	AR Perrault syndrome POI-Infertility & SNHL	LE	5.91
NUP107	mRNA transport & translation	AR Ovarian dysgenesis 6	LE	2.37
		**Candidate genes for POI**		
PTEN	AKT/PKB signalling inhibition	Candidate. AD Cowden disease	0.1	<1
SMC3	Cohesin complex	Candidate. AD Cornelia de Lange type 3	0.51	4.44
MCMBP	Double strand break repair	Candidate	0.62	25.30
ERCC5	DNA mismatch repair	Candidate. AR XPGC	0.53	6.21
MEI1	Meiotic double strand breaks	Candidate. AR Hydatidiform Mole	0.35	<1
		**Documented genes for neuropathic diseases**		
HARS1	Ligation of histidine to tRNA (protein biosynthesis)	AD CMT2W / AR USH3B	0.23	9.31
GARS1	Ligation of glycine to tRNA (protein biosynthesis)	AD CMT2D / AD SMAJI	0.42	22.60
SPG11	Vesicle trafficking	AR CMT2X / AR ALS5 /<br>AR SPG11	1.16	<1

Analyses were done as previously reported (ref 15). Only the genes detected at sufficient levels to allow comparisons of cases and controls for at least one of those U12sr/U2sr or U12ret/FPKM were given.

U12sr: Spliced reads across two exons joined by U12-type processing

U2sr: Spliced reads across two exons joined by U2-type processing

U12ret: Reads within a non-spliced U12-type intron

FPKM: Fragments Per Kilobase of transcript per Million fragments mapped

ca/co ratio: ratio of the specific values in cases (n=2) versus controls (n=4)

LE: Low expression levels

AKT/PKB: Protein Kinase B

AR: Autosomal recessive

AD: Autosomal dominant

CMT2W: Charcot-Marie-Tooth disease type 2W

CMT2D: Charcot-Marie-Tooth disease type 2D

SMAJI: Spinal Muscular Atrophy, James type, Infantile

SPG11: Spastic Paraplegia 11

SNHL: Sensorineural hearing loss

USH3B: Usher syndrome type 3B

XPGC: Xeroderma Pigmentosum, Group G

ALS5: Amyotrophic Lateral Sclerosis 5, juvenile

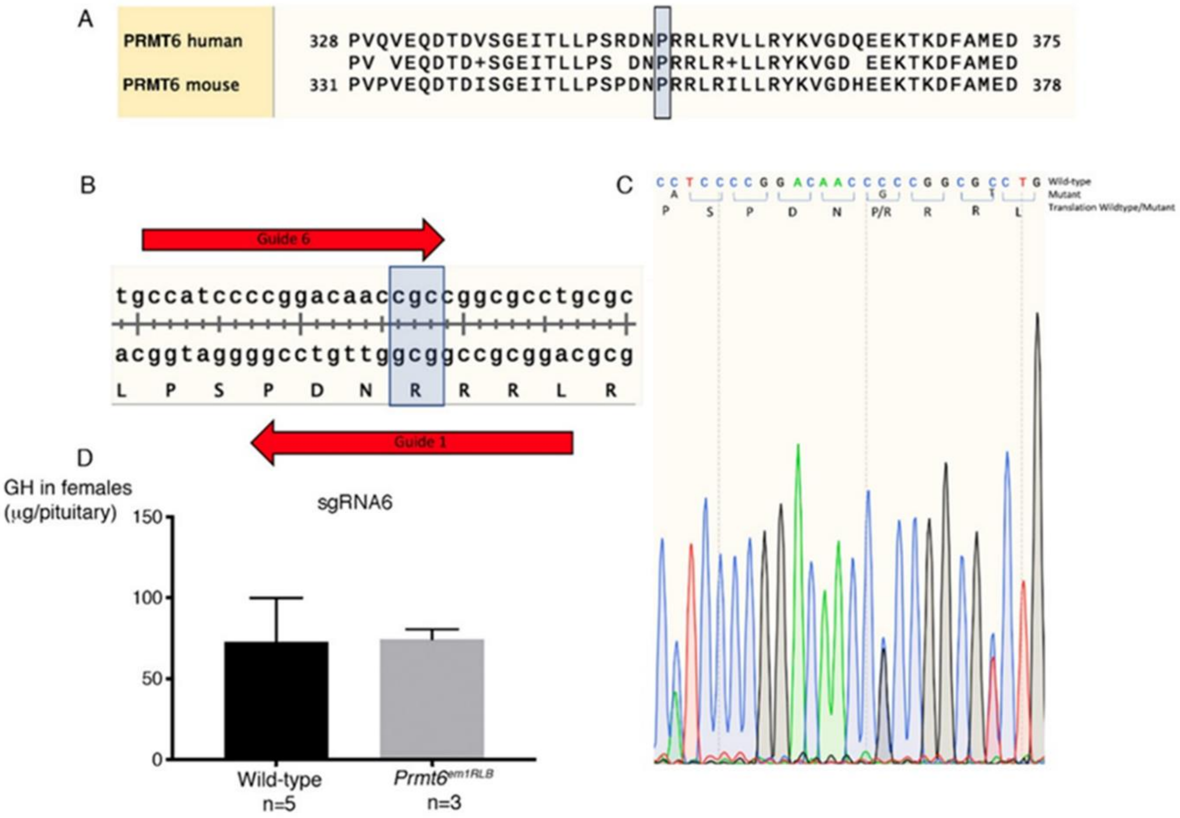

Figure S1:

A) Alignment of the mouse and human PRMT6 protein sequences with the consensus in the middle. The proline residue at position 350 that is the position of the pathogenic variant in patients and the surrounding region are conserved between mouse and human. + denotes similar residues.

B) Two single guide RNAs were designed where the proline codon (ccc) was changed to arginine (cgc).

C) Chromatogram of a mouse heterozygous *Prmt6* mutant sanger sequencing showing changed nucleotides giving rise to the desired missense variant (P to R) and the introduction of two synonymous variants to avoid subsequent cutting by the sgRNA.

D) RIA performed on female pituitaries comparing homozygous mutants and wild-type controls do not show any difference.

A

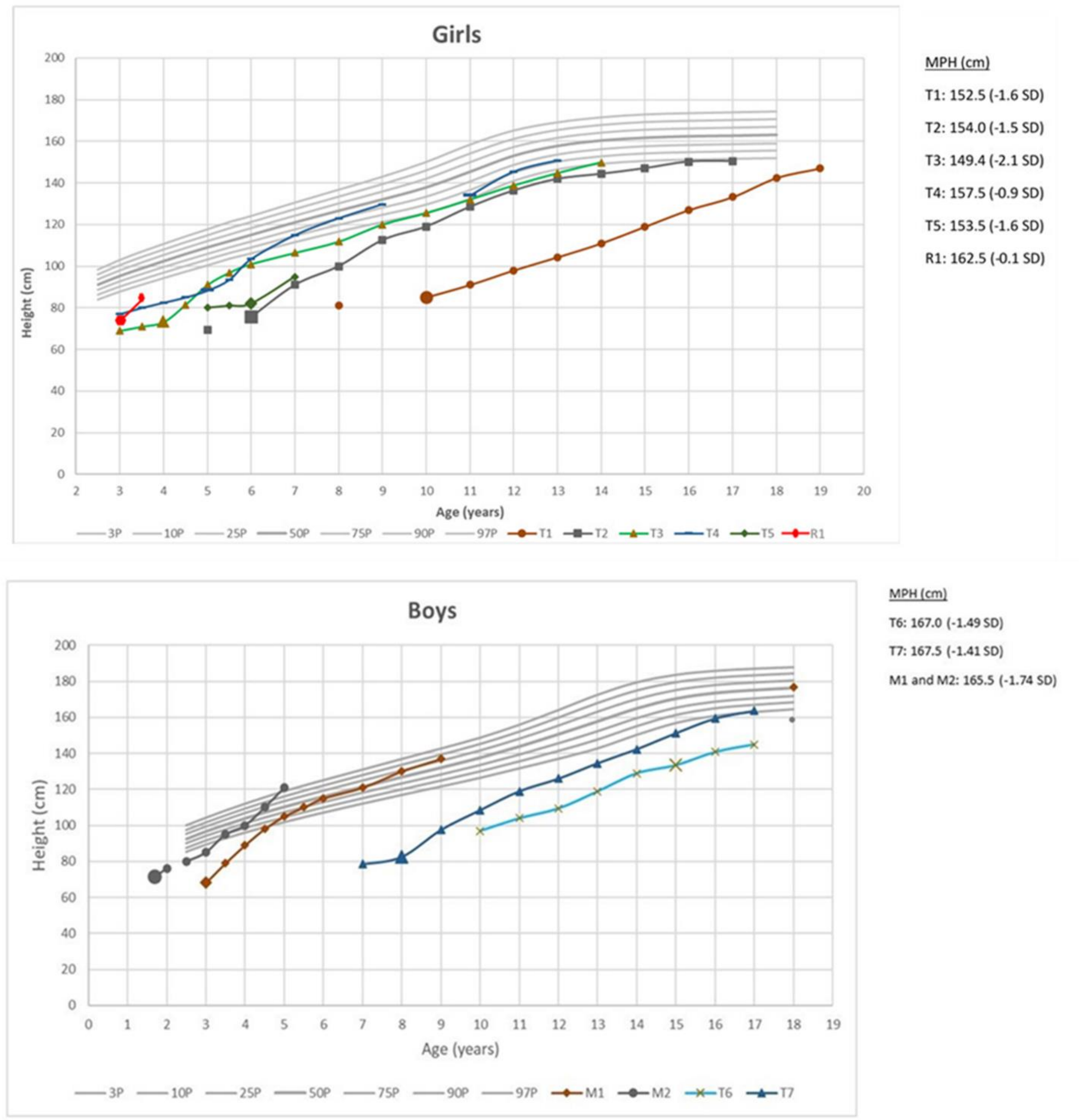

Figure S2-A: Growth charts showing the 3rd, 10th, 25th, 50th, 75th, 90th and 97th centile for height according to updated Turkish normative data. Patients' data are plotted. The points where rHGH treatment started are indicated with a bigger size marker for each patient. For patient T4, the treatment was ceased for 21 months during which growth rate decreased to 0.5 cm/year and was restarted at age 10.9 years. Patient T5 was lost to follow up after 1-year of treatment. Patient T6 showed growth without GH during the pubertal ages, rHGH treatment was started at age 15 years, but he was unsuccessfully in reaching his target adult height. Patient M1 and M2 stopped rHGH treatment at the age of 9.5 years and 5.3 years, respectively. They continued to grow without GH with patient M1 reaching 176.7 cm and patient M2 reaching 158 cm at adult height. Mid-parental height (MPH) for each patient is shown on the right side of the curve.

B

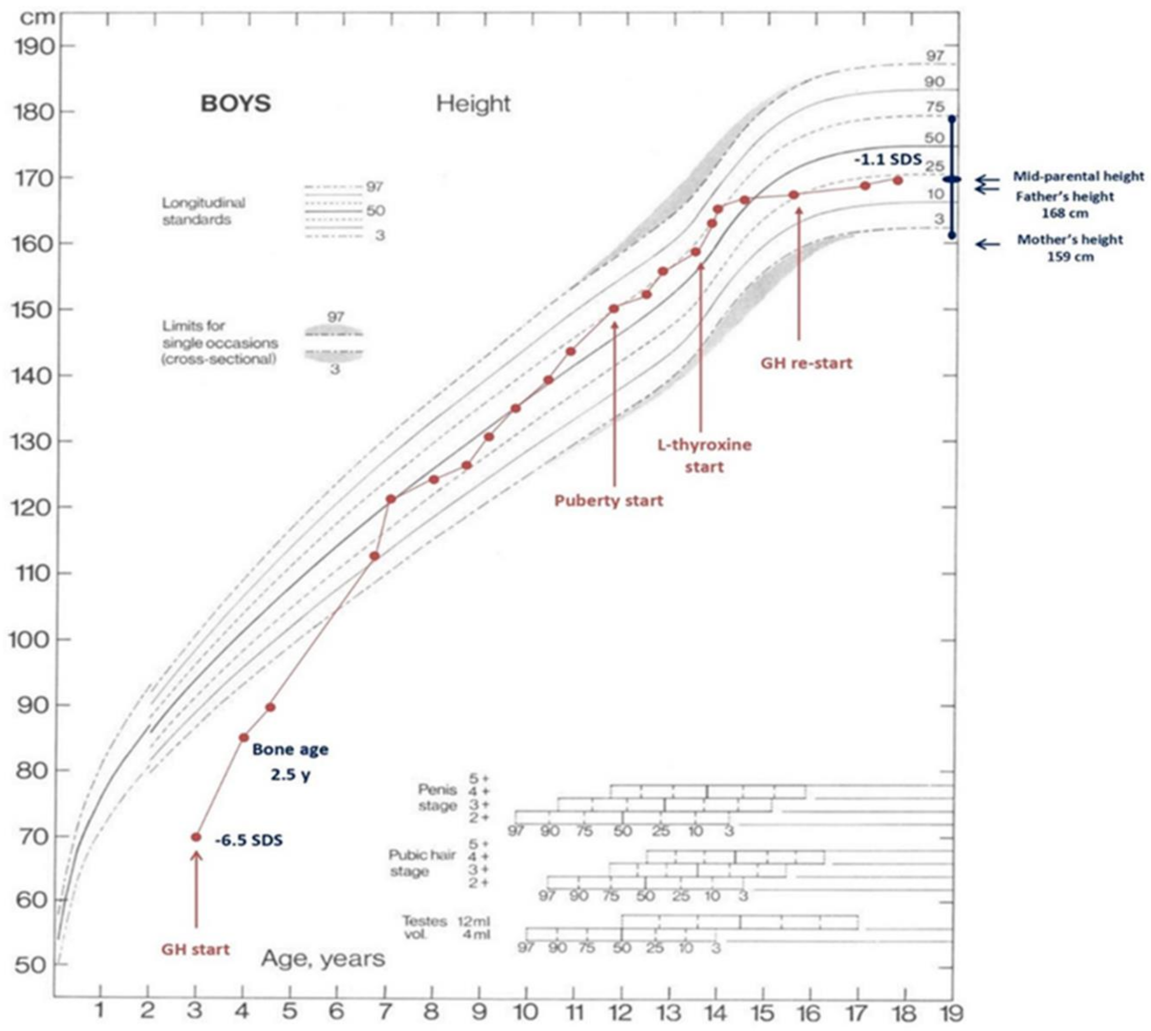

Figure S2-B: Growth chart of the patient I1. The points where rHGH treatment, puberty and L-treatment started are indicated with an arrow. rHGH was stopped for 3 months at age 15.7 years and restarted thereafter. Mid-parental height is shown on the right side of the curve.

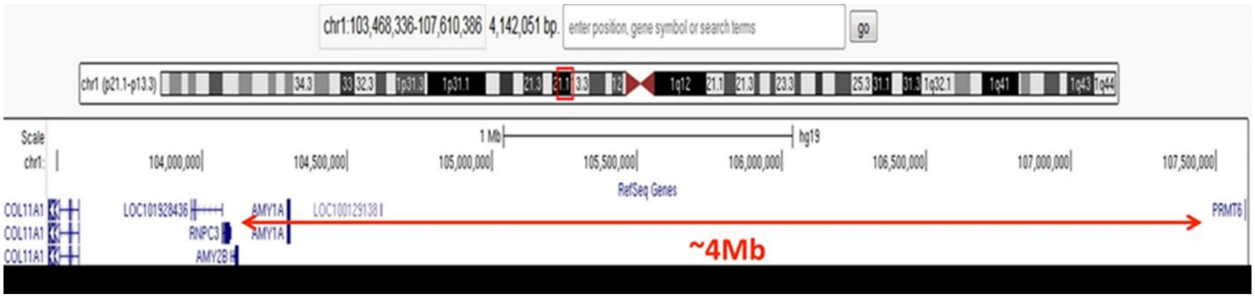

Figure S3: The genes *RNPC3* and *PRMT6* are located in close proximity to each other in the genome.

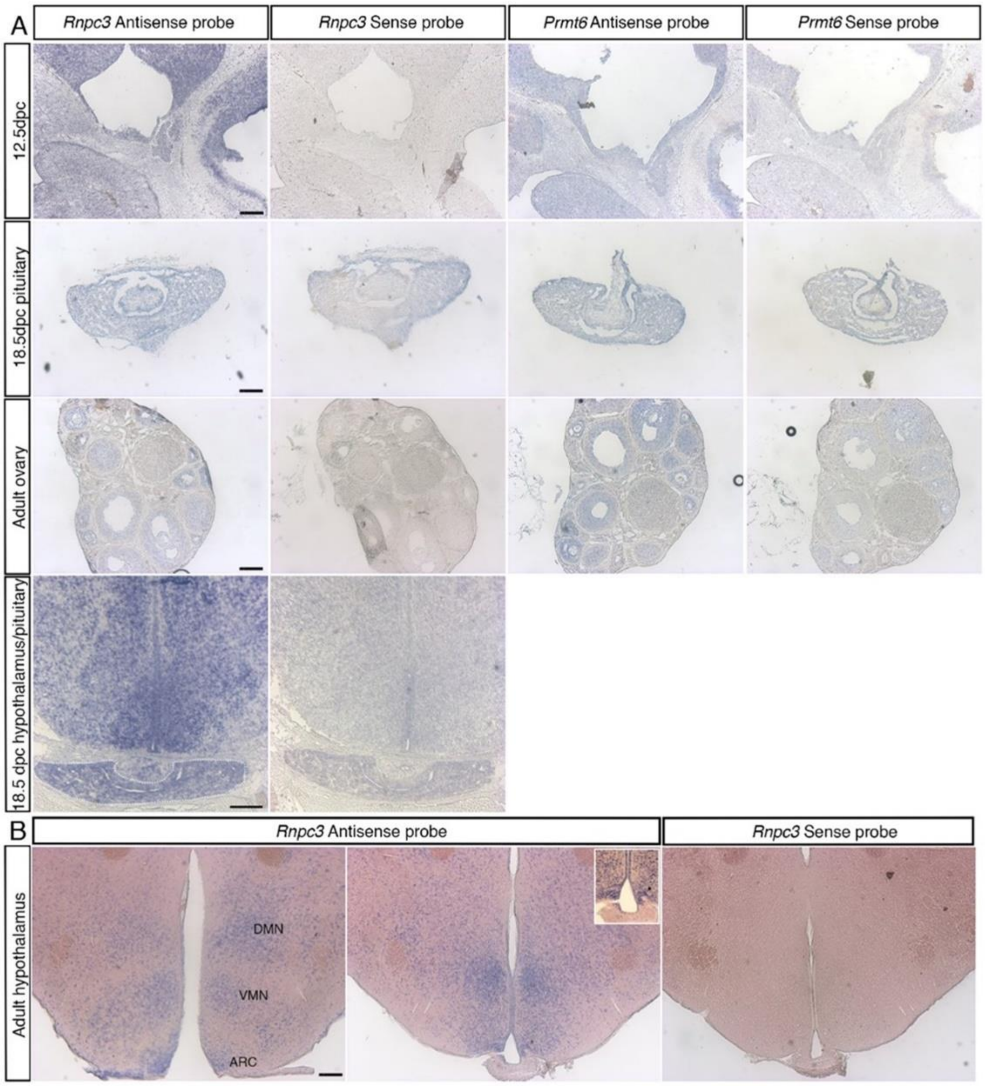

Figure S4: Expression of *Rnpc3* and *Prmt6* in the ovary and during mouse hypothalamo-pituitary axis development

A) Expression of *Rnpc3* and *Prmt6* was examined by *in situ* hybridisation. *Rnpc3* and *Prmt6* are both expressed in the developing brain and Rathke's pouch (outlined) as shown in sagittal sections at 12.5dpc. Expression is maintained in the pituitary at 18.5dpc. Both genes are also expressed in the adult ovary, predominantly in granulosa cells. *Rnpc3* is strongly expressed in the hypothalamus and anterior pituitary (outlined) at 18.5dpc while expression of *Prmt6* is weaker at this stage (data not shown).
B) Expression of *Rnpc3* was further examined in the adult hypothalamus where medial hypothalamic nuclei showed expression. In addition, tanycytes and/or ependymocytes flanking the third ventricle (inset) are *Rnpc3* positive.
Staining specificity is confirmed by lack of, or weak background staining observed in section hybridised with sense probes.
DMN: dorso-medial nucleus. VMN: ventro-medial nucleus. ARC: arcuate nucleus.
In A, the scale bars represent 150 mm for the 12.5dpc panel, and 100 mm for the others. In B it represents 50 mm.

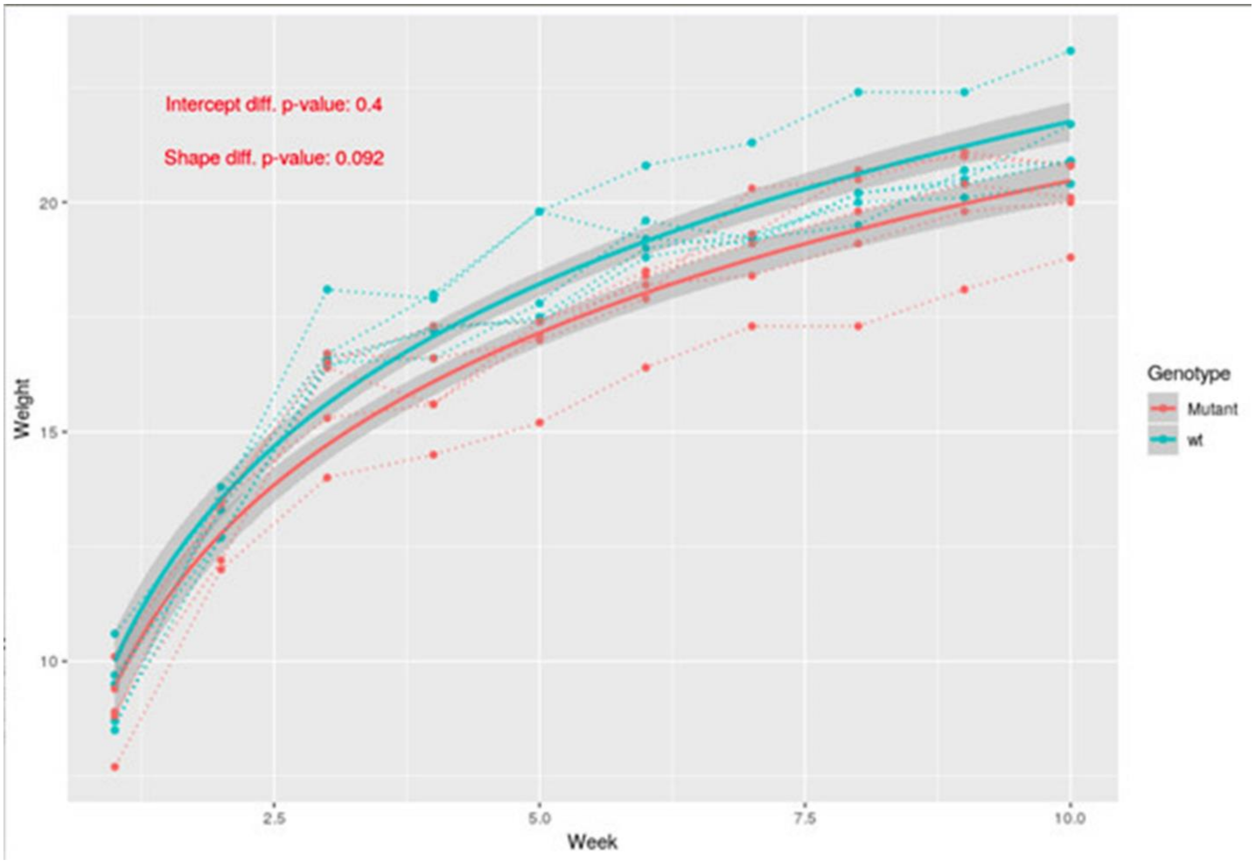

Figure S5: Growth curves of wild-type and homozygous sgRNA1 p.Leu483Phe female littermates. Females were weighted once/week from 1 to 10-week-old. Regression of the weight against time was performed to assess the effect of the *Rnpc3* alteration. While the mean curve of mutant female weight is below that of control, the difference did reach not statistical significance (intercept difference, ns). Furthermore, the animal weight gain pattern during growth is similar in both genotypes (shape difference, ns). This is in agreement with the limited reduction in GH observed by RIA in mutant females (Fig 2).

References

1 Li H, Durbin R. Fast and accurate short read alignment with Burrows-Wheeler transform. *Bioinformatics*. 2009;25:1754-60.

2 McKenna A, Hanna M, Banks E et al. The Genome Analysis Toolkit: A MapReduce framework for analyzing next-generation DNA sequencing data. *Genome Res*. 2010;20:1297-1303.

3 Wang K, Li M, Hakonarson H. ANNOVAR: functional annotation of genetic variants from high-throughput sequencing data. *Nucleic Acids Res.* 2010;38:e164.

4 Adzhubei IA, Schmidt S, Peshkin L, et al. A method and server for predicting damaging missense mutations. *Nat Methods* 2010;7:248-249.

5 Ng PC, Henikoff S. Predicting deleterious amino acid substitutions. *Genome Res*. 2001;11:863-874.

6 Argente J, Flores R, Gutiérrez-Arumí A, et al. Defective minor spliceosome mRNA processing results in isolated familial growth hormone deficiency. *EMBO Mol Med*. 2014;6:299–306.

7 Abou Tayoun AN, Pesaran T, DiStefano MT, Oza A, Rehm HL, Biesecker LG, Harrison SM; ClinGen Sequence Variant Interpretation Working Group (ClinGen SVI). Recommendations for interpreting the loss of function PVS1 ACMG/AMP variant criterion. Hum Mutat. 2018;39:1517-1524.

8 Rizzoti K, Brunelli S, Carmignac D, Thomas PQ, Robinson IC, Lovell-Badge R. SOX3 is required during the formation of the hypothalamo-pituitary axis. *Nat Genet*. 2004;36:247-55.8

9 Gregory LC. Investigation of new candidate genes in a cohort of patients with familial congenital hypopituitarism and associated disorders. Doctoral thesis, URI:1541141. University College London, 2017.

10 Gonen N, Futtner CR, Wood S, et al. Sex reversal following deletion of a single distal enhancer of Sox9. *Science*. 2018;360:1469–1473.

11 Wang C, Catlin DH, Demers LM, Starcevic B, Swerdloff RS. Measurement of total serum testosterone in adult men: comparison of current laboratory methods versus liquid chromatography-tandem mass spectrometry. *J Clin Endocrinol Metab.* 2004;89:534-43.

12 Gilligan LA, Trout AT, Schuster JG, Schwartz BI, Breech LL, Zhang B, Towbin AJ. Normative values for ultrasound measurements of the female pelvic organs throughout childhood and adolescence. *Pediatr Radiol*. 2019;49:1042-1050. 5

13 European Society for Human Reproduction and Embryology (ESHRE) Guideline Group on POI *et al.*ESHRE Guideline: management of women with premature ovarian insufficiency. *Hum. Reprod.* 2016;31, 926–937.
